# Novel Corneal Protein Biomarker Candidates Reveal Iron Metabolic Disturbance in High Myopia Eyes

**DOI:** 10.3389/fcell.2021.689917

**Published:** 2021-10-01

**Authors:** Jingyi Chen, Wenjing Wu, Zhiqian Wang, Chuannan Zhai, Baocheng Deng, Mohammad Alzogool, Yan Wang

**Affiliations:** ^1^School of Medicine, NanKai University, Tianjin, China; ^2^Tianjin Key Lab of Ophthalmology and Visual Science, Tianjin Eye Hospital, Tianjin Eye Institute, Nankai University Eye Hospital, Tianjin, China; ^3^Department of Optometry, Shenyang Eye Institute, The 4th People’s Hospital of Shenyang, Shenyang, China; ^4^Department of Cardiology, Tianjin Chest Hospital, Tianjin, China; ^5^Department of Infectious Disease, The 1st Affiliated Hospital of China Medical University, Shenyang, China

**Keywords:** myopia, cornea, protein biomarkers, signal, iron metabolism, protein–protein interaction, oxidative stress

## Abstract

Myopia is a major public health concern with increasing global prevalence and is the leading cause of vision loss and complications. The potential role of the cornea, a substantial component of refractive power and the protective fortress of the eye, has been underestimated in the development of myopia. Our study acquired corneal stroma tissues from myopic patients undergoing femtosecond laser-assisted small incision lenticule extraction (SMILE) surgery and investigated the differential expression of circulating proteins between subjects with low and high myopia by means of high-throughput proteomic approaches—the quantitative tandem mass tag (TMT) labeling method and parallel reaction monitoring (PRM) validation. Across all corneal stroma tissue samples, a total of 2,455 proteins were identified qualitatively and quantitatively, 103 of which were differentially expressed between those with low and high myopia. The differentially abundant proteins (DAPs) between the groups of stroma samples mostly demonstrated catalytic activity and molecular function regulator and transporter activity and participated in metabolic processes, biological regulation, response to stimulus, and so forth. Pathway enrichment showed that mineral absorption, ferroptosis, and HIF-1 signaling pathways were activated in the human myopic cornea. Furthermore, TMT analysis and PRM validation revealed that the expression of ferritin light chain (FTL, P02792) and ferritin heavy chain (FTH1, P02794) was negatively associated with myopia development, while the expression of serotransferrin (TF, P02787) was positively related to myopia status. Overall, our results indicated that subjects with low and high myopia could have different proteomic profiles or signatures in the cornea. These findings revealed disturbances in iron metabolism and corneal oxidative stress in the more myopic eyes. Iron metabolic proteins could serve as an essential modulator in the pathogenesis of myopia.

## Introduction

Myopia, as a complex multifactorial disease, is a globally recognized epidemic and a common cause of vision impairment characterized by its increasing prevalence among younger generations and heterogeneity among regions and ethnicities (Holden et al., 2016). Moreover, high myopia also foreshadows the irreversible visual damage caused by its pathological complications, such as retinal detachment, myopia-related retinopathy, and choroidal neovascularization (Wong et al., 2014).

To date, the underlying pathogenesis of myopia is still elusive, and various theories have been proposed to explain its development. The projected mechanisms for myopia are universally considered to be triggered by a combination of genetic susceptibility and environmental elements, among which metabolic factors are the most intricate, with little implications from the available evolutionary analyses (Morgan et al., 2012). Previous practices have attempted to address the impact of retinal defocus, which triggers the retina–choroid pathway, or hypoxia of the sclera, which would cause scleral collagen remodeling (Morgan et al., 2018). The associated mechanisms have been predominantly studied in animal models. Steeper corneas have been implicated in high amounts of form-deprived myopia in experimental animal models (Qiao-Grider et al., 2010). Furthermore, several manipulations justified alterations of the anterior and posterior segments in mammalian and avian ametropia models (Troilo et al., 2019). Nonetheless, the potential roles of the cornea, representing a substantial component of refractive power, and the protective fortress of the eyeball have been underestimated in myopic studies. High-quality analyses conducted in regions with the highest prevalence of myopia could provide useful information using representative clinical ocular samples.

Recent omics studies on myopia have predominantly focused on genomics, in order to identify genetic risk factors. Various international and polycentric genome-wide association studies (GWAS) and meta-analyses have been conducted on refractive phenotypes (Hysi et al., 2014). Proteomic analyses of myopia have hitherto included serum, tear, and aqueous humor samples from humans and retinal, scleral, and vitreous humor samples from animal models, considering sample availability and visually guided structural alterations during model establishment (Grochowski et al., 2020). A recent study first reported differentially abundant corneal proteins in high myopia chick models (Kang et al., 2021). However, no biomarker that corresponds to the diagnosis and classification of human myopia has yet been identified. The exploration of novel biomarkers would provide additional information on disease pathogenesis and related pathways. Therefore, a more comprehensive understanding of the molecular functions and processes involved in the development of high myopia is necessary to promote these interventions.

Small incision lenticule extraction (SMILE) surgery takes advantage of a femtosecond laser to shape a refractive lenticule from the corneal stroma, which is then removed through a particular small incision (Kim et al., 2019). The present study aimed to characterize the potential molecular pattern of the distinct evolution of low versus high myopia in patients scheduled to undergo SMILE surgery for myopia.

## Materials and Methods

### Phase 1: Exploratory Study: Proteomic Approaches to Biomarker Discovery

#### Subjects Enrollment

The current study acquired approval from the Institutional Review Board of Nankai University and Nankai University Eye Hospital (Ethics Number 201922) and abided by the tenets of the World Medical Association Declaration of Helsinki. The study was case-controlled and cross-sectional and conformed to the Strengthening the Reporting of Observational Studies in Epidemiology (STROBE) guidelines of observational studies. All enrolled participants agreed to the sample collection, and informed consent was obtained. In total, 62 systemically healthy subjects aged 18–34 years (96 eyes), were recruited between 2019 and 2020. Complete general and ophthalmic histories were collected from all participants. Since this study aimed at a proteomic evaluation for low versus high myopia eyes, an individual eye was set as a target, rather than an individual subject, which meant both eyes could be selected from the same subject. The enrolled eyes were divided into the low-myopia and high-myopia groups. The group stratification in our study was determined with respect to the spherical equivalent (SE), which was assessed as sphere + cylinder/2. The SE was defined as less than –3.00D for low myopia and over –6.00D for high myopia (Morgan et al., 2012). The inclusion criteria were as follows: generalized myopic patients scheduled for SMILE surgery with written informed consent; absence of a history or examination evidence of ocular trauma; and absence of unrelated ocular diseases such as cataract, glaucoma, or retinopathy. The systemic exclusion criteria were the presence of systemic disorders such as diabetes mellitus, respiratory or cardiovascular diseases, hypertension, kidney disease, severe infection status, conditions of inflammation or current pregnancy, and not taking any medication such as antimetabolites, immunosuppressants, or steroids.

#### Sample Collection

Among the 96 eyes of 62 participants, 18—9 eyes with low myopia and 9 eyes with high myopia, respectively, were randomly selected for sample collection. Corneal stroma tissue samples were acquired during VisuMax (Carl Zeiss Meditec, Jena, Germany) SMILE surgery at Nankai University Eye Hospital. Each stroma sample was kept separately in an Eppendorf tube, marked, and frozen in liquid nitrogen immediately after the lenticule extraction procedure. The samples were then stored at –80°C until measurement.

#### Protein Extraction: Homogenate and SDT Lysis

The nine corneal stroma samples in either group were randomly mixed into three sample mixtures for the tandem mass tag (TMT) proteomic experiment and future validation study. Three mixed samples from low or high myopia eyes were randomly set as one biological parallel (Huang et al., 2020). An equal batch of adequate corneal stroma tissue from each sample was amalgamated and pulverized into a powder. The sorted samples were then homogenized by adding SDT1 buffer [4% sodium dodecyl sulfate (SDS), 1 mmol dithiothreitol (DTT), 100 mmol Tris–HCl, pH 7.6] and transferred to Eppendorf tubes with quartz sand (MP homogenizer, 24 × 2, 6.0 M/S, 60 s, twice). Homogenate of protein extraction was carried out by sonication (power 80 W, worktime 10 s, interval 10 s, cycle 10 times) on ice, and the extractions were then boiled for 15 min. The crude digest was centrifuged for 40 min at 14,000 *g*, and the supernatant was filtered through 0.22 μm filters. The filtrate was calculated using a BCA Protein Assay Kit (Bio-Rad, United States), and the collection was stored at –80°C for future measurement. Sodium dodecyl sulfate polyacrylamide gel electrophoresis (SDS-PAGE) protein separation was performed. Protein samples (20 μg) were mixed in 5 × loading buffer and boiled for 5 min. Protein separation was performed using a 12.5% SDS-PAGE gel (with 14 mA constant current for 90 min). Coomassie Blue R-250 staining was used to visualize the acquired protein bands (Zhu et al., 2014).

#### Filter-Aided Sample Preparation Protein Digestion

Protein digestion was performed using filter-aided sample preparation. Briefly, 200 μg of total protein from each sample was incorporated into SDT2 buffer (4% SDS, 100 mmol DTT, 150 mmol Tris–HCl, pH 8.0), boiled for 5 min, and then cooled to 25°C. Excessive DTT, detergents, and other low-molecular-weight components in the protein samples were washed out using UA buffer (8 M urea, 150 mmol Tris–HCl, pH 8.0) by repeated ultrafiltration (Microcon units, 10 kD, 14,000 *g*, 15 min). Each filter extract was then added to 100 mM iodoacetamide to inhibit the reduced cysteine residues. The extracts were incubated for 30 min in the dark and then centrifuged for 40 min at 14,000 *g*. The filtrate was discarded, and the filter was washed in 100 μl UA buffer three times and then in 100 μl of 100 mM triethylammonium bicarbonate (TEAB) buffer twice. Subsequently, 4 μg trypsin (Promega, United States) was dissolved in 40 μl TEAB buffer, and protein suspension was added to this buffer and digested overnight at 37°C. Finally, the digested samples were centrifuged to harvest the resulting peptides, and the concentrations of peptide contents were calculated according to 280 nm UV light spectral density by means of an extinction coefficient of 1.1 in 0.1% (g/L) solution measured in the light frequency of tyrosine and tryptophan in the vertebrate protein atlas (Wiśniewski et al., 2009).

#### Tandem Mass Tag Labeling and High pH Reversed-Phase Fractionation

Each consequent peptide mixture (100 μg) was labeled with TMT reagent (Thermo Fisher Scientific, United States). Principally, the three mixed samples of low myopia were labeled with 126, 127C, and 127N isobaric TMT tags, and the three mixed samples of high myopia were labeled with 129C, 130C, and 130N isobaric TMT tags. After TMT labeling, the labeled digest samples were fractionated, and 10 fractions were obtained. The surplus labels and salts were diminished using a high reversed-phase fractionation kit (Thermo Fisher Scientific, United States) to increase the acetonitrile step-gradient elution.

#### Liquid Chromatography–Mass Spectrometry/Mass Spectrometry Analysis

Liquid chromatography–mass spectrometry/mass spectrometry (LC-MS/MS) analysis was performed for each fraction. The peptide compounds were dissolved in buffer A (0.1% formic acid) and then loaded onto a reverse-phase trap column (Thermo Fisher Scientific, PepMap100, 100 μm × 2 cm, nano Viper C18) which was coupled to a C18 reverse-phase analytical column (Thermo Scientific, 75 μm × 10 cm, 3 μm resin) and divided using linear-gradient buffer B (84% acetonitrile + 0.1% formic acid) at a flow rate of 300 nl/min. The linear gradient was processed using the following parameters: 0–55% buffer B for 80 min, 55–100% buffer B for 5 min, and 100% buffer B for 5 min.

LC-MS/MS was performed on a Q Exactive mass spectrometer connected to Easy nLC (Thermo Fisher Scientific) in positive ion mode for 90 min. The procedure was performed in peptide recognition mode. MS data were obtained using a data-dependent top 10 method, which allows dynamic selection of the most copious precursor ions from the 300 to 1,800 m/z survey scan for higher-energy collisional dissociation (HCD) fragmentation. The instrument parameters were set as follows: automatic gain control (AGC) target, 3e6; HCD spectra, 35,000 resolution at m/z 200; survey scans. Seventy thousand resolution at m/z 200; width of resolution, 2 m/z; maximum injection time, 10 ms; and dynamic exclusion duration, 40.0 s. The normalized collision energy was set to 30 eV, and the underfill ratio was defined as 0.1% to allow the minimum percentage of the target value to be accomplished at maximum full time.

#### Data Analysis and Protein Identification

The acquired LC-MS/MS spectra were searched using the MASCOT engine (version 2.2; Matrix Science, London, United Kingdom) in Proteome Discoverer 1.4 (Thermo Electron, San Jose, CA, United States). The following parameters were selected: the enzyme applied was trypsin, fragment mass tolerance of 0.1 Da, peptide mass tolerance of ± 20 ppm, and maximum missed cleavages of 2. Fixed modifications were set for the TMT-10 plex, and variable modifications were applied for oxidation. The false discovery rate of the peptides was set as < 0.01. Protein quantification was calculated using protein ratios, which were presented as the median of protein unique peptides. The median protein ratio was used to normalize all peptide ratios. The median protein ratio was defined as 1 after normalization for the experimental bias. The D’Agostino and Pearson normality test was used to calculate data normality. Chi-square and Student’s *t*-tests were used to analyze demographic and clinical data. For quantitative protein expression analysis, pair-wise group comparisons were performed using LC-MS, and significant differences between groups were assessed using normalized protein abundances in arcsinh transformation. To distinguish differentially abundant proteins (DAPs), the fold change was set as > 1.2 or < 0.83. A *p*-value (Student’s *t*-test) of < 0.05 was considered statistically significant (Li et al., 2019).

#### Bioinformatic Analysis

##### Hierarchical Clustering Analysis

Hierarchical clustering analysis was performed using protein relative expression data via Cluster 3.0 software^1^ and Java Treeview software^2^. The Euclidean distance algorithm for similarity measure and average linkage clustering algorithm using centroids of the observations for clustering were selected. In addition to a dendrogram, a heatmap is often presented as a visual aid. The clustering heatmap and volcano plots of DAPs were visualized using R 3.6.0 software (R: A Language and Environment for Statistical Computing, R Development Core Team).

##### Gene Ontology and Kyoto Encyclopedia of Genes and Genomes Pathway Annotations

The expression trends of human corneal stromal proteins were searched in the UniProt KB database and retrieved in FASTA format in batches. The retrieved sequences of differentially expressed proteins were searched with reference to the local SwissProt database (human) using NCBI BLAST + client software for homolog sequences from which the gene ontology (GO) functional annotation could be merged. In this procedure, the top 10 blast hits with *E*-values of <1e^–3^ for every query sequence were restored and loaded into Blast2GO software (Version 3.3.5) (Götz et al., 2008) for GO prognostication and annotation, which was set with default gradual enzyme code (EC) weights, a GO weight of 5, an annotation cutoff of 75, and a filtered *E*-value of 1e^–6^. The unannotated sequences without BLAST hits were re-annotated with more permissive parameters and were then selected to go through InterProScan (Quevillon et al., 2005) meriting comparison with European Bioinformatics Institute (EBI) databases to fetch functional annotations of protein motifs and derive the InterProScan GO annotation terms. The results of the GO annotation sets were mapped using R scripts (R Development Core Team).

The protein sequences in FASTA form of differentially expressed proteins were searched in the Kyoto Encyclopedia of Genes and Genomes (KEGG) database^3^. The corresponding KEGG pathways were retrieved and extracted (Moriya et al., 2007).

##### Functional Enrichment Analysis

Functional enrichment analysis was carried out to further explore the influence of differentially altered proteins in cellular physio-pathological processes and the discovery of internal connections between the DAPs. The entire protein quantification dataset was set as the background. GO term enrichment on three ontology modules (BP—biological process, MF—molecular function, and CC—cellular component) and KEGG pathway annotation analyses were measured using Fisher’s exact test. The derived *p*-values were further adjusted by the application of Benjamin–Hochberg correction for multiple tests, and only GO functional categories and KEGG pathways with *p*-values < 0.05 were considered statistically significant.

##### Protein–Protein Interaction Network

The information of protein–protein interaction (PPI) of the target proteins was searched using the gene symbols and retrieved from the STRING (Search Tool for the Retrieval of Interacting Genes/Proteins) online database^4^ or IntAct molecular interaction database^5^. The results were downloaded and imported into Cytoscape software (version 3.2.1)^6^. Visualization and functional analysis of the PPI networks were conducted. Additionally, the degree of interaction of each target protein was calculated to assess its significance in the PPI network.

### Phase 2: Validation Study: Parallel Reaction Monitoring Analysis

#### Parallel Reaction Monitoring Assay Development

##### Prioritized Target Protein Selection

The next step after the TMT discovery experiment involved an LC-parallel reaction monitoring (PRM)-based workflow to establish sensitive and accurate detection of the relative abundance of the candidate protein markers. Considering the scarcity of clinically available corneal stroma samples, the PRM validation study was carried out using the same cohort in the phase 1 exploratory study to reinforce the consistency between studies. However, reagent costs have limited the possibility of performing analyses for all identified candidate proteins, which necessitates a further step of prioritization. As a result, the identified proteins from the phase 1 TMT experiment were coped with hierarchical clustering, GO functional enrichment, KEGG pathway enrichment, and PPI network analysis for a comprehensive overview of the DAPs between the comparisons.

##### Peptide Selection

In the next step of targeted PRM assays, the selected proteins were optimized in corneal stroma samples using a set of three proteo-unique peptides. The unique peptides were screened from the phase 1 study. For proteins with fewer than three proteo-unique peptides, extra peptides were searched through the online selected reaction monitoring (SRM) Atlas^7^ (Bostanci et al., 2018). All designated peptides ranged from 6 to 20 amino acids in length and contained tryptic ends without any missing cleavages. For the PRM analysis, SPOT synthesis (JPT Peptide Technologies, Germany) was applied to standard stable isotope-labeled peptides that were identical to the proteo-typic peptides and contained either a C-terminal arginine or lysine residue in unpurified form for chemical synthesis.

##### Sample Preparation and Protein Digestion

The protein expression levels obtained by TMT analysis were further quantified by LC-PRM/MS analysis (Peterson et al., 2012). A portion of 200 μg proteins from each sample was designated for in-solution trypsin digestion following the TMT protocol, and an aqua-stable isotope peptide was spiked per sample as an internal standard reference. Tryptic peptides were loaded on C18 stage tips (Wicom International AG, Maienfeld, Switzerland) for desalting prior to reversed-phase liquid chromatography (RPLC) on the Easy nLC-1200 system (Thermo Fisher Scientific). We proceeded with LC for 1 h with gradients of acetonitrile ranging from 5 to 35% in 45 min.

##### Parallel Reaction Monitoring Measurements

The succeeding PRM measurements were continued on a Q Exactive Plus mass spectrometer (Thermo Scientific). Highly intensive proteo-unique peptides were utilized for the confidential analysis of each target protein with optimization for charge state, collision time, and retention times. The mass spectrometer was operated in positive ion mode. The following parameters were set: full MS1 scan resolution, 70,000 (at 200 m/z); AGC target, 3.0 × 10^–6^; and ion injection time, 200 ms maximum. After the full MS scans, 20 PRM scans were followed at 35,000 resolution (at 200 m/z) with AGC 3.0 × 10^–6^ and 200 ms maximum injection times. The resulting peptides were consequently isolated in a 2Th window with ion activation/dissociation at a collision energy of 27 within a higher energy dissociation collision cell (Zhang et al., 2019).

#### Parallel Reaction Monitoring Data Processing

The PRM raw data were processed using the Skyline bioinformatics tool (MacCoss Lab, University of Washington, United States) (MacLean et al., 2010) where the detected signal intensities for individual peptide sequences of each target protein were quantified with standard reference normalization and relative to the respective sample. The Skyline PRM acquisition methods were time-scheduled, and the quantification files contained the results from all selected samples with measurements of the target proteins, including three target proteins and five unique peptide sequences, both heavy and light. A proper *Q*-value was selected to filter the results of peptide assays for high data quality, where a *Q*-value > 0.05 was removed. In addition, assays with reported 0 intensity were removed. Subsequently, the light-to-heavy ratio was programmed as log2 fold change. The correlation between peptide transitions was computed and demanded a quality filter. All peptide transitions with more than 10 nucleic acids and with a Pearson correlation of <0.5 were removed from the dataset. The median of the log2(l/h) ratios for peptide transitions was used to acquire peptide quantification and to obtain the protein log2(l/h) ratios.

#### Predictive Protein–Protein Interaction Analysis

The STRING functional protein association networks online database (see text footnote 4) and GeneMANIA online database for genes and gene sets functional predictions^8^ were used for the critical evaluation and integration of predictive PPI networks based on the PRM validation evidence as well as genomic and proteomic knowledge gained from human studies. The prioritized proteins were mapped individually and integrally to create the predicted images.

## Results

### Phase 1: Discovery Study—Tandem Mass Tag Proteomic Approach for Quantitative Analysis

#### Characteristics of the Study Subjects

The present study included 96 eyes of 62 systemically healthy subjects, among which 18 eyes from 13 subjects were randomly selected for proteomic discovery analysis. The low myopia group included nine eyes (OD = 4/OS = 5) from seven subjects (six males/one female), aged 24 ± 6.5 years, with an SE of –1.94 ± 0.30D. The high myopia group included nine eyes (OD = 5/OS = 4) from six subjects (five males/one female), aged 22.6 ± 6.8 years, with an SE of –8.68 ± 0.60D. No significant differences were noted between the two groups in terms of age, gender, and left or right eye selected, but subjects in the low myopia group had a significantly lower SE than did those in the high myopia group (*p* < 0.001).

#### Protein Identification and Differentially Abundant Protein Analysis

**FIGURE 1:**
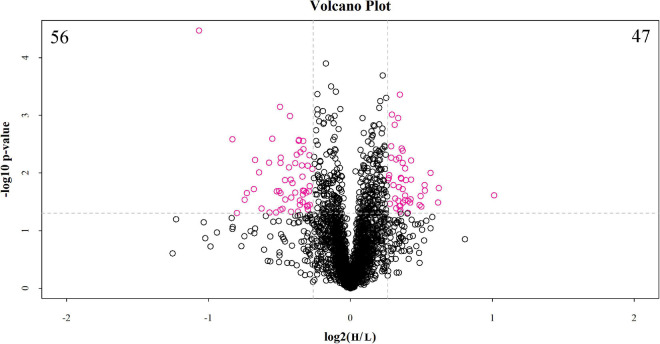
Volcano plot of differentially abundant proteins between the low and high myopia groups. The volcano plot shows a significant difference in differentially abundant proteins (DAPs) between the two groups of samples. The x-axis represents the difference multiple (log2 fold change), and the y-axis represents the *p*-value of the difference (–log10). The red dots in the figure represent the significant DAPs (multiple changes > 1.2 or < 0.83 and *p* < 0.05), and the black dots show that there are no differences detected in the proteins between the two groups. H, high myopia group; L, low myopia group.

**FIGURE 2:**
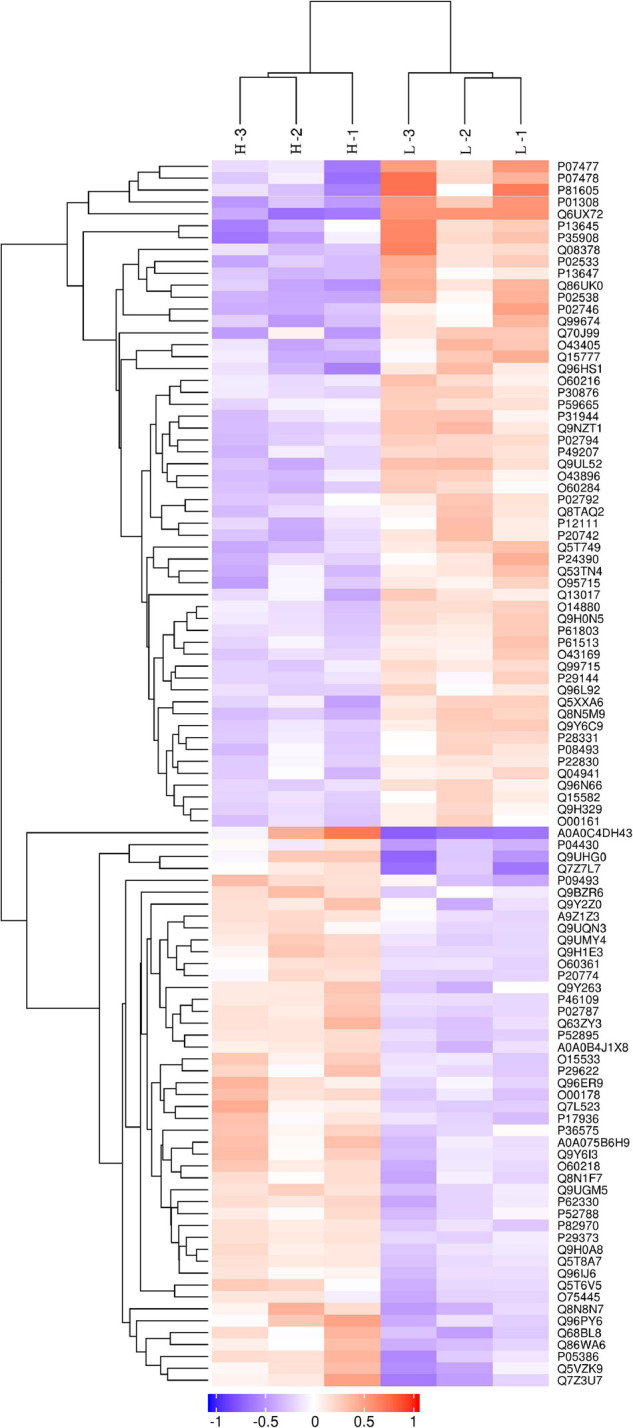
Clustering analysis of differentially abundant proteins. Hierarchical clustering analysis was performed using a tree heat map. In the heat map, each row represents a protein [i.e., the ordinate represents the significant differentially abundant proteins (DAPs)], and each column represents a group of samples (the abscissa is the sample information). The logarithm values (log2 expression) of the significant DAPs are displayed in different colors, where red represents significantly upregulated proteins, purple represents significantly downregulated proteins, and gray represents no available protein quantitative information.

The discovery study was conducted using high-throughput TMT quantitative proteomic techniques. A total of 2,455 different proteins in the *Homo sapiens* protein atlas were identified (Supplementary Table 1). Overall, 103 DAPs were confirmed in the comparison between low and high myopic eyes. Among the DAPs, 47 proteins were upregulated, and 56 proteins were downregulated (Figure 1). A heat map was plotted for the visualization of the hierarchical clustering analysis of the identified DAPs (Figure 2).

#### Bioinformatic Analysis and Functional Enrichment Analysis

The results of the bioinformatics and functional enrichment analyses are presented in Table 1. The significantly altered proteins between the low-myopia and high-myopia groups were further categorized, whereby relevant GO and KEGG term enrichment were prioritized with regard to their *E*-value and *p*-value. The mapped GO function and KEGG pathway demonstrated that the functions of the DAPs were predominantly catalytic activity, binding, molecular function regulator, structural molecule activity, and transporter activity, and the most significantly regulated processes in high myopia compared with low myopia were “metabolic process,” “cellular process,” “biological regulation,” “response to stimulus,” and “regulation of biological process.”

**TABLE 1:** Top five ontology terms for GO and KEGG analysis.

Ontology	Description	Adjust *p*-value	RichFactor
BP	Keratinization	<0.001	0.27
BP	Cornification	<0.001	0.25
BP	Epidermis development	<0.001	0.15
BP	Keratinocyte migration	0.001	0.5
BP	Epidermal cell differentiation	0.001	0.15
MF	Ferric iron binding	<0.001	1
MF	Ferrous iron binding	<0.001	0.57
MF	Oxidoreductase activity	<0.001	0.6
MF	Structural constitute of cytoskeleton	0.005	0.15
MF	Ferroxidase activity	0.005	0.67
CC	Keratin filament	<0.001	0.56
CC	Intermediate filament	<0.001	0.21
CC	Intracellular ferritin complex	0.002	1
CC	Ferritin complex	0.002	1
CC	Intermediate filament cytoskeleton	0.003	0.17
KEGG	Mineral absorption	<0.001	0.5
KEGG	Protein digestion and absorption	0.014	0.17
KEGG	Staphylococcus aureus infection	0.018	0.16
KEGG	Ferroptosis	0.026	0.19
KEGG	Viral protein interaction with cytokine	0.042	1

*GO, gene ontology; KEGG, Kyoto Encyclopedia of Genes and Genomes; BP, biological process; MF, molecular function; CC, cellular component.*

**FIGURE 3:**
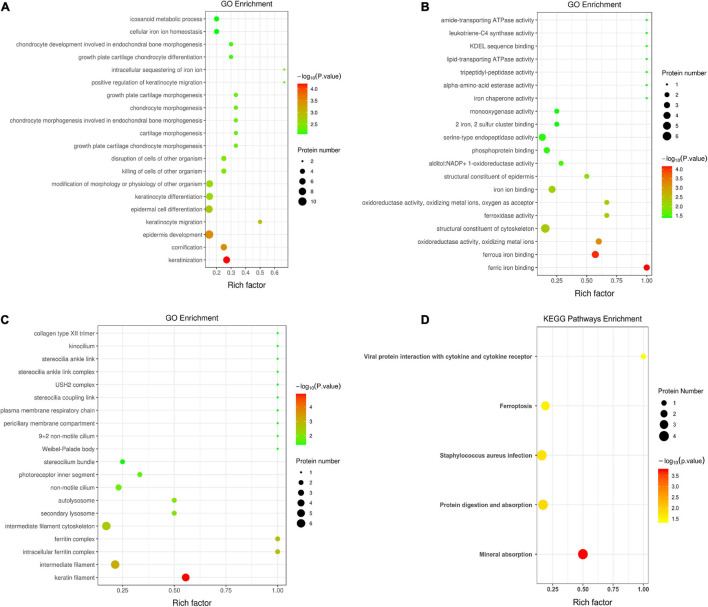
Gene ontology annotation and Kyoto Encyclopedia of Gene and Genomes pathway enrichment analysis between the low and high myopia groups. The ordinate in the figure stands for the enriched gene ontology (GO) functional annotation, which can be divided into **(A)** BP, **(B)** MF, and **(C)** CC or **(D)** enriched Kyoto Encyclopedia of Genes and Genomes (KEGG) pathways. The abscissa stands for enrichment factors (rich factor ≤ 1). The rich factor represents the proportion of DAPs annotated in a functional ontology to the number of all identified proteins annotated in that functional ontology. The size of the bubbles in the figure indicates the number of differentially expressed proteins in each classified functional ontology. The color of the bubbles indicates the significance of the enriched functional categories. The color gradient displays the *p*-value, where the closer to red, the smaller the *p*-value and the higher the significance level of the corresponding ontology.

**FIGURE 4:**
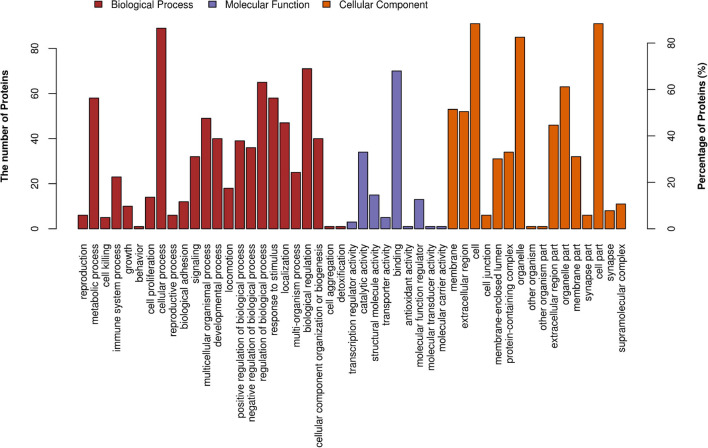
GO level 2 functional analysis. The x-axis shows the enriched GO level 2 functional annotation, which was demonstrated as BP (red), MF (purple), and CC (orange). The y-axis shows the number and percentage of proteins detected. BP, biological process; MF, molecular function; CC, cellular component.

Figure 3 demonstrates the top 20 enriched GO terms of BP (Figure 3A), MF (Figure 3B), and CC (Figure 3C). The main biological processes of DAPs involved metabolic process, cellular process, biological regulation, and response to stimulus. The main molecular functions were associated with ferric iron binding, ferrous iron binding, structural molecule activity, catalytic activity, and molecular transducer activity. The main cellular components of these proteins were exhibited as cell and organelle part, ferritin complex, intracellular ferritin complex, and protein-containing complex (Figure 4).

**FIGURE 5:**
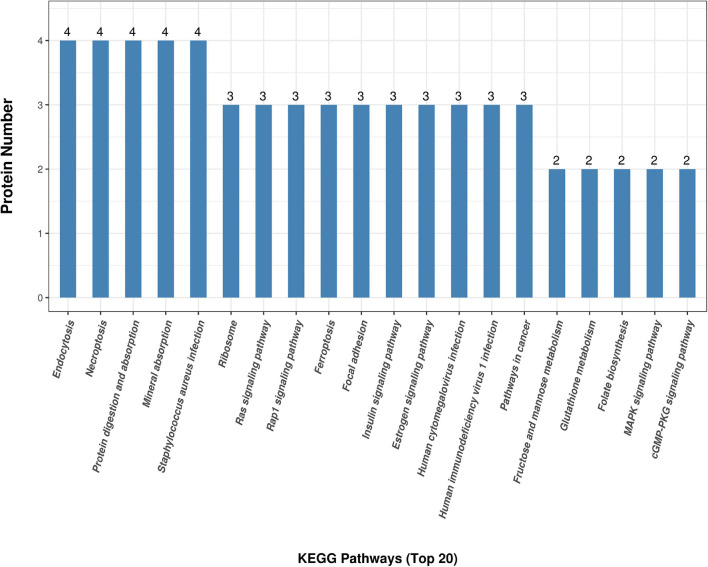
KEGG top 20 pathway enrichment. The x-axis demonstrates the top 20 KEGG enriched pathways. The y-axis demonstrates the number of proteins participating in the enriched pathways.

Figure 3D shows the KEGG pathway enrichment analysis. The DAPs were enriched into mineral absorption, ferroptosis, staphylococcus aureus infection, protein digestion, and absorption pathways. Figure 5 demonstrates the top 20 enriched KEGG pathways.

**FIGURE 6:**
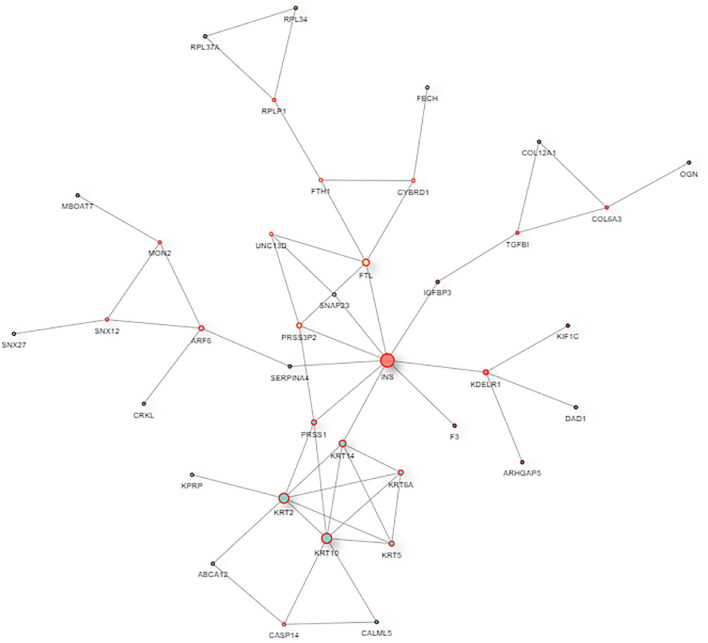
The protein–protein interaction network of DAPs. In the protein–protein interaction (PPI) network, the colored nodes stand for differentially altered proteins, and lines are mapped for the interactions between proteins. Larger nodes correspond to a higher degree of protein aggregation in the PPI network.

The interaction map of the differentially expressed proteins, which were enriched in a larger interaction network, is presented in Figure 6. PPI analysis suggested that the development of myopia could result from the dysfunction of multiple pathways.

### Phase 2: Validation Study—Verification of Myopia-Associated Protein Biomarker Candidates via the PRM Approach

#### Protein Selection and Parallel Reaction Monitoring Measurements

**FIGURE 7:**
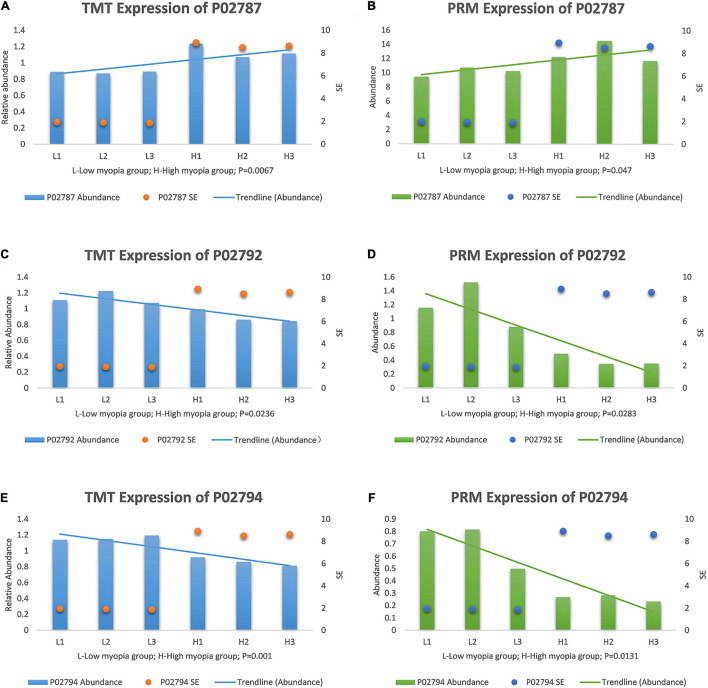
Expression patterns of target protein biomarker candidates using tandem mass tag (TMT) analysis and parallel reaction monitoring (PRM) validation with their expression trends compared with SE, **(A)** TMT expression of serotransferrin (P02787). **(B)** PRM expression of P02787. **(C)** TMT expression of ferritin light chain (P02792). **(D)** PRM expression of P02792. **(E)** TMT expression of protein ferritin heavy chain (P02794). **(F)** PRM expression of P02794.

After a thorough evaluation of the GO annotation, KEGG pathway analysis, and PPI network analysis, we focused on the main GO terms “ferric iron binding” and “oxidizing metal irons” and the main KEGG pathways “mineral absorption” and “ferroptosis.” Therefore, we further selected three iron- and redox-related DAPs, including serotransferrin (TF, P02787), ferritin light chain (FTL, P02792), and ferritin heavy chain (FTH1, P02794) for validation studies using the PRM approach. TF is a major iron-uptake protein. Ferritin is a major iron-storage protein. Among the validation results, the abundance of TF was increased in the high myopia group, while FTL and FTH1 expression were both reduced in the high myopia group compared to the low myopia group (Figure 7). It could be deduced from the PRM results that the selected proteins presented semblable trends, similar to the aforementioned TMT proteomic results. Verification of the candidate DAPs confirmed the credibility of our proteomic research.

#### Predictive Protein–Protein Interaction Analysis

**FIGURE 8:**
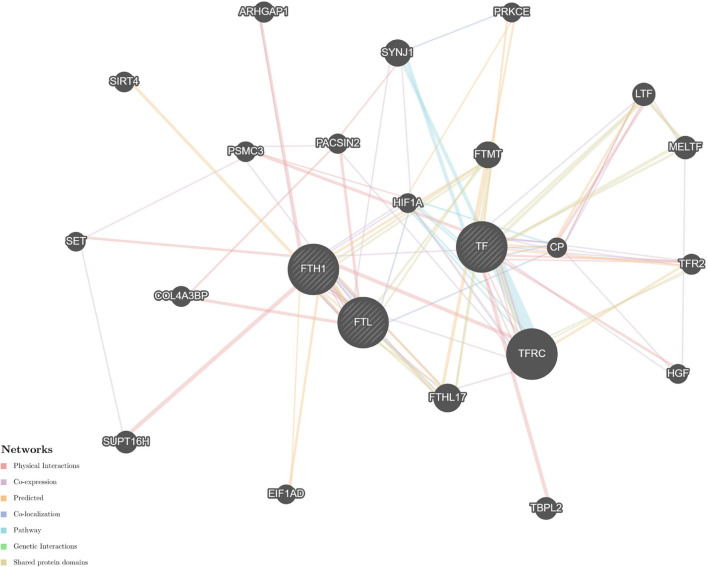
Predictive PPI network of the validated protein biomarker candidates: TF-FTL-FTH1. In the PPI network prediction, the nodes in the middle with bias represent the validated protein biomarkers, and lines are mapped for the interactions between proteins. Larger nodes correspond to a higher degree of protein aggregation in the PPI network, and different colors correspond to various functional networks.

**FIGURE 9:**
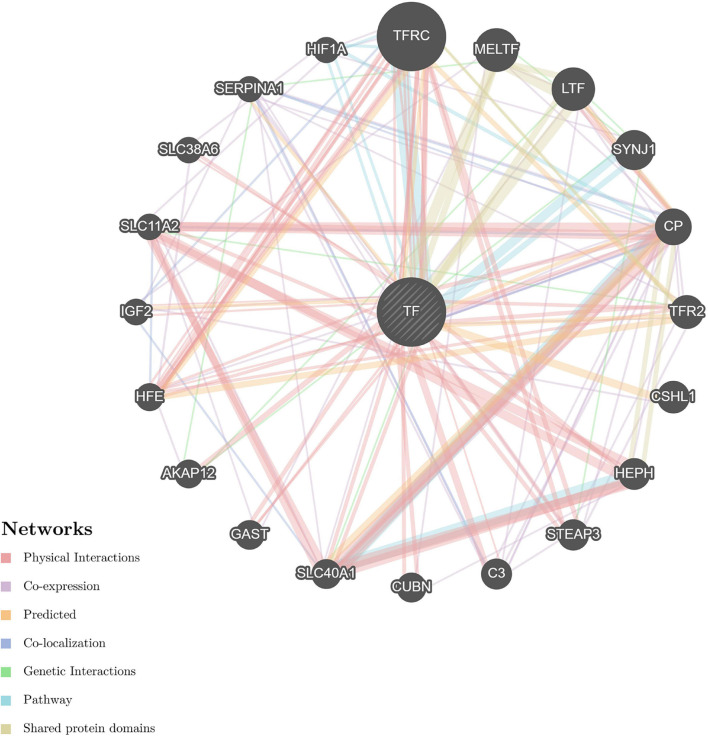
Predictive PPI network of the target protein biomarker candidates: TF. In the PPI network prediction, the node in the middle with bias represents the target protein biomarker, and lines are mapped for the interactions between proteins. Larger nodes correspond to a higher degree of protein aggregation in the PPI network, and different colors correspond to various functional networks.

**FIGURE 10:**
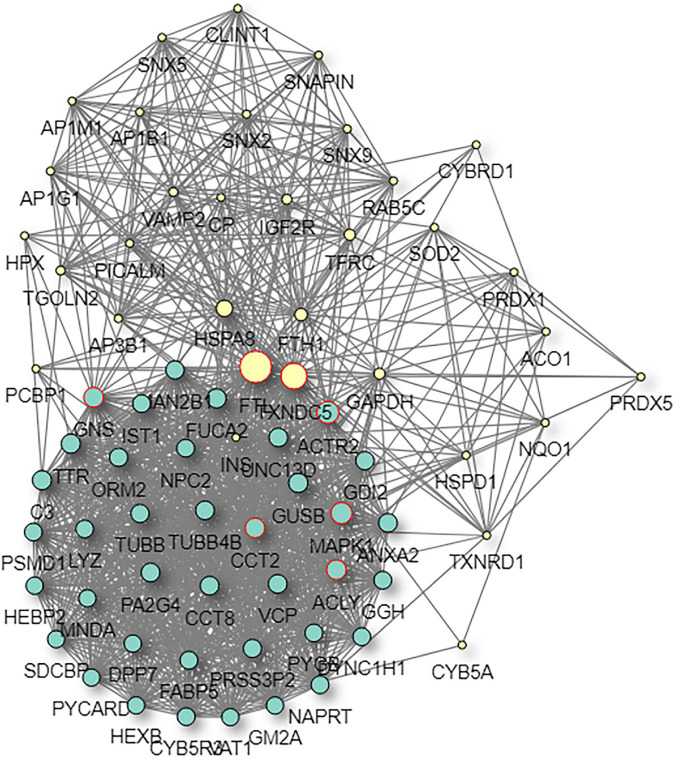
Predictive PPI network of the target protein biomarker candidate: FTL. In the PPI network prediction, the colored node in the middle represents the target protein biomarker, and lines are mapped for the interactions between proteins. Larger nodes correspond to a higher degree of protein aggregation in the PPI network.

**FIGURE 11:**
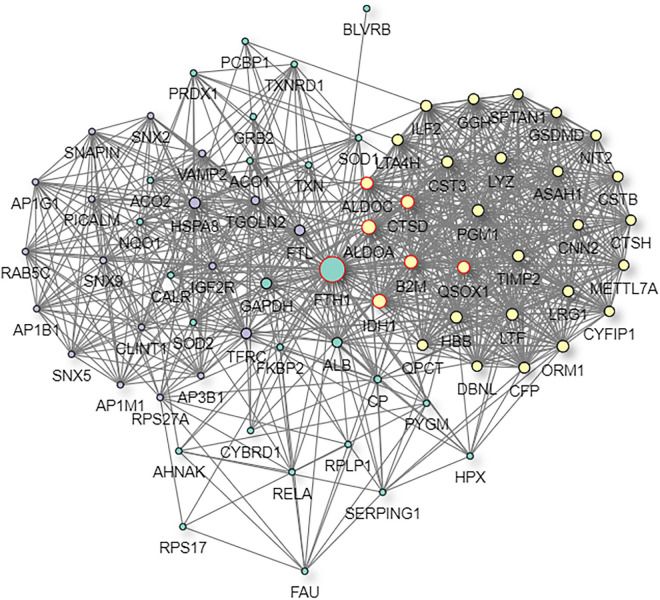
Predictive PPI network of the target protein biomarker candidate: FTH1. In the PPI network prediction, the colored node in the middle represents the target protein biomarker, and lines are mapped for the interactions between proteins. Larger nodes correspond to a higher degree of protein aggregation in the PPI network.

The STRING and GeneMANIA online tools were further applied for the integration and prediction analysis of PPIs of the validated target proteins. The prioritized proteins—TF, FTL, and FTH1—were mapped, and an integrated predicting PPI network image was created (Figures 8–11 and Supplementary Figure 1).

## Discussion

In this study, for the first time, we employed proteomic approaches to investigate the molecular characterization of the human corneal stroma in relation to myopia. The present study has uncovered the broadest human corneal stroma proteome to date, as compared with previous studies (Dyrlund et al., 2012), and discovered different proteomic signatures between low and high myopia eyes. The stroma proteome provides novel biomarker candidates and signaling pathways to further elucidate the mechanisms underlying myopia development.

The pending issue of the complex pathogenesis and lack of adequate treatment has made the prevention and control of myopia difficult. Moreover, the available approaches for differentiating low and high myopia rely primarily on ocular biometric parameters, including SE and axial length. Therefore, the identification of potential molecular patterns is essential for further understanding the mechanism and susceptibility of myopia and offer new perspectives for early diagnosis and therapeutic targets.

Generally speaking, GWAS are valuable in myopia-associated research but may not succeed in addressing a large portion of genetic variability. Analysis of gene–gene interaction networks and regulation of signaling pathways, rather than studying genes individually, has greater potential for investigating related phenotypes (Lauwen et al., 2017). In the past decade, human proteomic studies have played an essential role in discerning the molecular mechanisms involved in the diagnosis and treatment of different diseases. The high stringency human proteome analysis developed targeted proteomic assays for the investigation of key proteins and signaling pathways (Adhikari et al., 2020). The novel tandem MS methods and PRM assays could permit the identification of peptides from various samples simultaneously according to their relative abundance with better performance, higher sensitivity, and greater accuracy than other proteomic methods (Thompson et al., 2003; Stergachis et al., 2011).

The DAP profiles and functional enrichment analyses of our study revealed novel iron metabolism features of myopic eyes, with implications for corneal reactive oxygen species (ROS) acceleration. The main GO annotations implicated the major biological processes “metabolic process” and “cellular iron ion homeostasis,” remarkable molecular functions of “ferric iron binding” and “ferroxidase activity,” and an outstanding cellular component of “ferric complex” (Figures 3A–C). The KEGG pathways were mainly enriched to “mineral absorption” and “ferroptosis” (Figure 3D). Furthermore, the three selected proteins (TF, FTL, and FTH1) associated with iron metabolism and oxidative stress exhibited a similar gradient between the TMT and PRM quantitative results.

Proteins play a significant role in ocular metabolism. The distribution of iron and iron homeostasis proteins in the rodent retina has been reported previously (Yefimova et al., 2000). Iron is an essential biometal and a principal source of nutrients for the eye. Previous studies characterized circulating iron and associated homeostasis proteins, such as ferritin and TF, in various ocular tissues, including the cornea (Linsenmayer et al., 2005), aqueous humor (Yu and Okamura, 1988), and lens (Harned et al., 2006).

TFs are iron-binding transport proteins responsible for heme absorption and utilization. TF binds two Fe^3+^ ions together with an anion, most commonly bicarbonate. They are growth factors required for all cells and play a central role in stimulating cell proliferation (Fulcher et al., 1988). It has been demonstrated that proliferating cells are capable of expressing TF receptors with enhanced density, making them preferential targets for the inhibition of malignant cells (Trowbridge and Domingo, 1981). The GO-biological process annotation implicates its role in cellular protein metabolic and iron homeostasis processes. To the best of our knowledge, this is the first study to illustrate the role of TF in human myopia, which, as an eye disorder, could present with hyperproliferative features. A previous animal quantitative proteomic analysis reported a remarkable upregulation of ovotransferrin, which belongs to the transferrin family in myopic chick vitreous. Ovotransferrin could function as an antioxidant in tissues, and its upregulation in the vitreous chamber indicates increased oxidative stress along with axial elongation (Yu et al., 2017). Future investigation of the mechanism of TF and TF receptors in corneal and other ocular cell divisions could offer further avenues for the study of proliferative conditions in myopic eyes.

Cellular iron is stored as cytosolic ferritin. Ferritins sequester intracellular iron in an innoxious and readily soluble form, which is vital for iron homeostasis. A ferritin molecule can hold up to 4,500 ferric-state iron molecules in its center core (Aisen et al., 2001). Iron is ingested in the ferrous form and deposited after oxidation as ferric hydroxides. Human ferritins consist of two types of ferritin subunits: H chain for heavy or heart and L chain for light or liver. A previous proteomic study by Karring et al. (2005) identified the presence of ferritin subunits in the human corneal epithelium. Free iron has the ability to catalyze ultraviolet-induced oxidation reactions via the Fenton reaction. FTH1 is a ferroxidase, and its increase is believed to reduce intracellular free iron oxidation levels and improve cellular resistance against oxidative stress. In contrast, FTL shares 50% identity with the FTH1 at the amino acid level. The light chain lacks the ferroxidase feature of the heavy chain but can facilitate iron cooperation within the ferritin cavity (Cozzi et al., 2000).

The intracellular iron status is registered by iron-regulatory proteins (IRPs). Regarding the status of intracellular iron deficiency, IRPs would bind to iron-responsive elements (IREs), which are present on the mRNAs of the regulated proteins. The IRE of ferritin lies on the 5′- terminal of ferritin mRNAs, and its binding with IRPs could efficiently obstruct the steric translation of ferritin, thereby inducing iron deficiency with insufficient ferritin levels. Reciprocally, the connection of IRPs with the IREs of transferrin and receptors, which lie on the 3′-portion of the mRNAs, would restrain the process of mRNA degradation, resulting in an increase in transferrin in iron deficiency (Rouault, 2002). In our proteomic study, the expression levels of FTL and FTH1 were both decreased in the high myopia group, while the TF abundance was increased in the high myopia group compared to the low myopia group, indicating the potential impact of iron deficiency on the progression of myopia.

Previous attempts to investigate ferritin metabolism in the cornea have been conducted in avian corneal epithelial cells, where FTH1 was observed to function as a developmentally regulated nuclear protein, although ferritin is one of the cytoplasmic components in most cells. The study revealed the similarity between the structure and biological properties of nuclear and cytoplasmic ferritin. Considering that corneal epithelial cells can be constantly exposed to ultraviolet light, nuclear ferritin is speculated to promote iron sequestration and prevent oxidative damage to cell DNA (Linsenmayer et al., 2005). Iron has been implicated in a wide range of ophthalmic disorders, such as cataracts, glaucoma, macular degeneration, and intraocular hemorrhage. However, the underlying correlation with myopia was underestimated. The derived PPI networks involved in iron metabolism are intriguing (Figures 8–11 and Supplementary Figure 1). Transferrin and ferritins are circulation proteins and are also considered essential cofactors in the integration of dopamine, neurotransmitters, and norepinephrine (He et al., 2007). Their alterations could suggest an environmental impact on the ocular surface and would in turn represent circulating features shown in the cornea.

Iron is crucial for various metabolic processes but can also cause oxidative stress by functioning as a reactive free radical. Iron ions are redox-active metals that can catalyze the production of OH^–^ from H_2_O_2_ (He et al., 2007). Oxidative stress can cause oxidative damage, resulting from the disequilibrium between free radical production and antioxidant defenses, which implicates the interaction of multiple molecular species. Oxidative stress has been described in previous reports of retinal and macular diseases with retinal pigment epithelium or choroidal atrophy. The cornea consumes oxygen mainly from oxygen in the air, and daily light exposure may have an impact on the cornea. Either of these processes could well generate ROS in the cornea (Francisco et al., 2015), which may induce DNA cleavage, protein alterations, and deleterious peroxidation of lipids. Hypoxia has been associated with various ophthalmic conditions caused by oxidative damage. This could be one of the key targets for myopia study since oxidative circumstances exist chronically (Wu et al., 2018).

Oxidative stress has been implicated in different ocular tissues in myopic eyes, such as the retina and sclera, which can explain the complex signaling pathways involved in the regulation of myopia, particularly the hypoxia-inducible factor-1 alpha (HIF-1α) signaling pathway. The TF gene was implicated in the HIF-1α signaling pathway according to a previous PPI network analysis (Wu et al., 2018). Hypoxia results in imbalanced cellular prooxidants and antioxidants via oxidative stress or ROS accumulation, which is a key mechanism of cytotoxicity (Junk et al., 2002). Oxidative stress leads to higher levels of HIF-1α, a subunit of the heterodimeric basic helix–loop–helix-structured HIF-1. HIF-1α protein is generally undetectable in well-oxygenated cells as it degrades rapidly. Under conditions of normoxia, prolyl hydroxylation is processed at its highly conserved prolyl residues by members of the prolyl hydroxylase domain family (PHD). Prolyl hydroxylases demand Fe^2+^, O_2_, ascorbate, and oxoglutarate for catalytic activity, and when HIF-1α is overexpressed, hydroxylation takes place and polyubiquitylation induces proteasomal degradation. PHD proteins belong to the Fe^2+^-dependent oxygenase superfamily. Conversely, the HIF-1α hydroxylation rate is suppressed under hypoxic conditions (Kaelin and Ratcliffe, 2008). Furthermore, the involvement of oxidative stress could implicate the process of chronic inflammation, leading to ocular tissue dysfunction during the progression of myopia.

Iron-associated corneal abnormalities are clinically manifest as corneal iron deposition. Investigations into such abnormalities have hitherto been conducted in physiological and pathological conditions (Yeung et al., 2006). Hudson–Stahli lines are typical in aging corneas, and a Fleischer ring is often suggestive of keratoconus (Gass, 1964). Other pathological conditions include a postoperative paracentral ring (Mannis, 1983) or central spot (Seiler and Holschbach, 1993) following ablative refractive surgery, and a fitting curve ring is observed in orthokeratology (Liang et al., 2003). The pathogenesis of corneal iron deposition has been an issue of major debate in recent decades. Both the epithelium basal cell migration theory and the combination theory of tear desiccation and senescent basal cell mechanism have been proposed (Assil et al., 1993). Previous observations have suggested that iron deposition is consistent with the area of greatest epithelial hyperplasia, which is promoted by basal cell mitosis and migration (Yeung et al., 2006).

Keratoectasia is considered the most devastating postoperative complication after ablative refractive surgeries because it causes severe vision loss. Fleisher’s ring is a typical sign of keratoconus, which represents corneal iron deposition in the epithelial basement membrane. Free iron in tissue can cause oxidative damage via Fenton and Haber-Weiss reactions that transfer hydrogen peroxide to free radicals. Ferritin expression is controlled by erythroid-derived 2. Ferritins block peroxide free radical formation and control the expression of nicotinamide adenine dinucleotide phosphate [NAD(P)H]: quinone oxidoreductase 1, which inhibits free radical formation by quinone redox cycling (Nioi and Hayes, 2004). Ferritin sequesters free iron, and the downregulated ferritin expression reported in corneas with keratoconus could explain the phenomenon of iron accumulation. Ferritins can protect cellular DNA from oxidative stress caused by free radicals or ultraviolet light (Joseph et al., 2011). The decrease in ferritin levels in the corneal stroma of myopic eyes implies reduced protective effects of the cornea and further increased oxidative damage in the cornea. Previous studies have implicated oxidative damage, metabolic malfunction, and increased cell death of corneal stromal keratocytes in keratoconus (Foster et al., 2014). The discovery of the associations among oxidative stress, alteration of iron metabolism, and myopia may offer further solutions for the identification of patients with potential keratectasia.

After reviewing a wealth of literature, we discovered potential clues between iron uptake and myopia. García-Casal and Leets (2014) investigated ferritin synthesis by Caco-2 cells and found that carotenoids—lycopene, lutein, and zeaxanthin—rather than vitamin A, could improve iron uptake from ferrous fumarate and NaFe-EDTA. Lutein is a major xanthophyll carotenoid found in the human retina at preferentially high concentrations. Various studies have reported its antioxidative and anti-inflammatory properties related to different ocular disorders, indicating a protective effect against oxidative and inflammatory diseases such as diabetic retinopathy, retinopathy of prematurity, and myopia. Furthermore, a significant inverse association was found between axial length and macular pigment levels in the Chinese adult population, including lutein and zeaxanthin (Li et al., 2020).

## Conclusion

Collectively, our study identified novel corneal protein biomarker candidates in high-myopia eyes, discovering different proteomic profiles between low and high myopia. The results further demonstrate that disturbances in iron homeostasis could be closely implicated in myopia development, and accelerated corneal oxidative stress was induced in the more myopic eyes. The final identification of rigorous biomarkers for high myopia requires further biological and functional experiments, as well as clinical studies. The key protein biomarker candidates identified and hallmark signaling pathways enriched in the study are under investigation to further determine their specific roles in the pathogenesis and development of myopia. Iron metabolism and associated proteins might serve as potential diagnostic or predictive biomarker candidates for high myopia and other conditions characterized by ocular oxidative damage.

## Data Availability Statement

The mass spectrometry proteomics data have been deposited to the ProteomeXchange Consortium via the PRIDE (Perez-Riverol et al., 2019) partner repository with the dataset identifier PXD025145.

## Ethics Statement

The studies involving human participants were reviewed and approved by the Ethical Committee of Nankai University. The patients/participants provided their written informed consent to participate in this study.

## Author Contributions

YW, JC, and WW designed the research, performed the research, and wrote the manuscript. JC, ZW, CZ, MA, and BD analyzed the data. JC, ZW, and CZ prepared the figures. JC, BD, and YW contributed analytic tools and edited the manuscript. JC and YW critically revised the manuscript and addressed feedbacks. YW acquired the funding. All authors contributed to the article and approved the submitted version.

## Conflict of Interest

The authors declare that the research was conducted in the absence of any commercial or financial relationships that could be construed as a potential conflict of interest.

## Publisher’s Note

All claims expressed in this article are solely those of the authors and do not necessarily represent those of their affiliated organizations, or those of the publisher, the editors and the reviewers. Any product that may be evaluated in this article, or claim that may be made by its manufacturer, is not guaranteed or endorsed by the publisher.

## Footnotes

1
http://bonsai.hgc.jp/~mdehoon/software/cluster/software.htm

2
http://jtreeview.sourceforge.net

3
https://www.kegg.jp/

4
http://string-db.org/

5
http://www.ebi.ac.uk/intact/

6
http://www.cytoscape.org/

7
http://www.srmatlas.org

8
http://genemania.org/
