# Supplementary figures and images for: Novel Corneal Protein Biomarker Candidates Reveal Iron Metabolic Disturbance in High Myopia Eyes
Source: Front Cell Dev Biol. 2021 Oct 1;9:689917. doi: 10.3389/fcell.2021.689917 (PMC8517150; doi:10.3389/fcell.2021.689917)

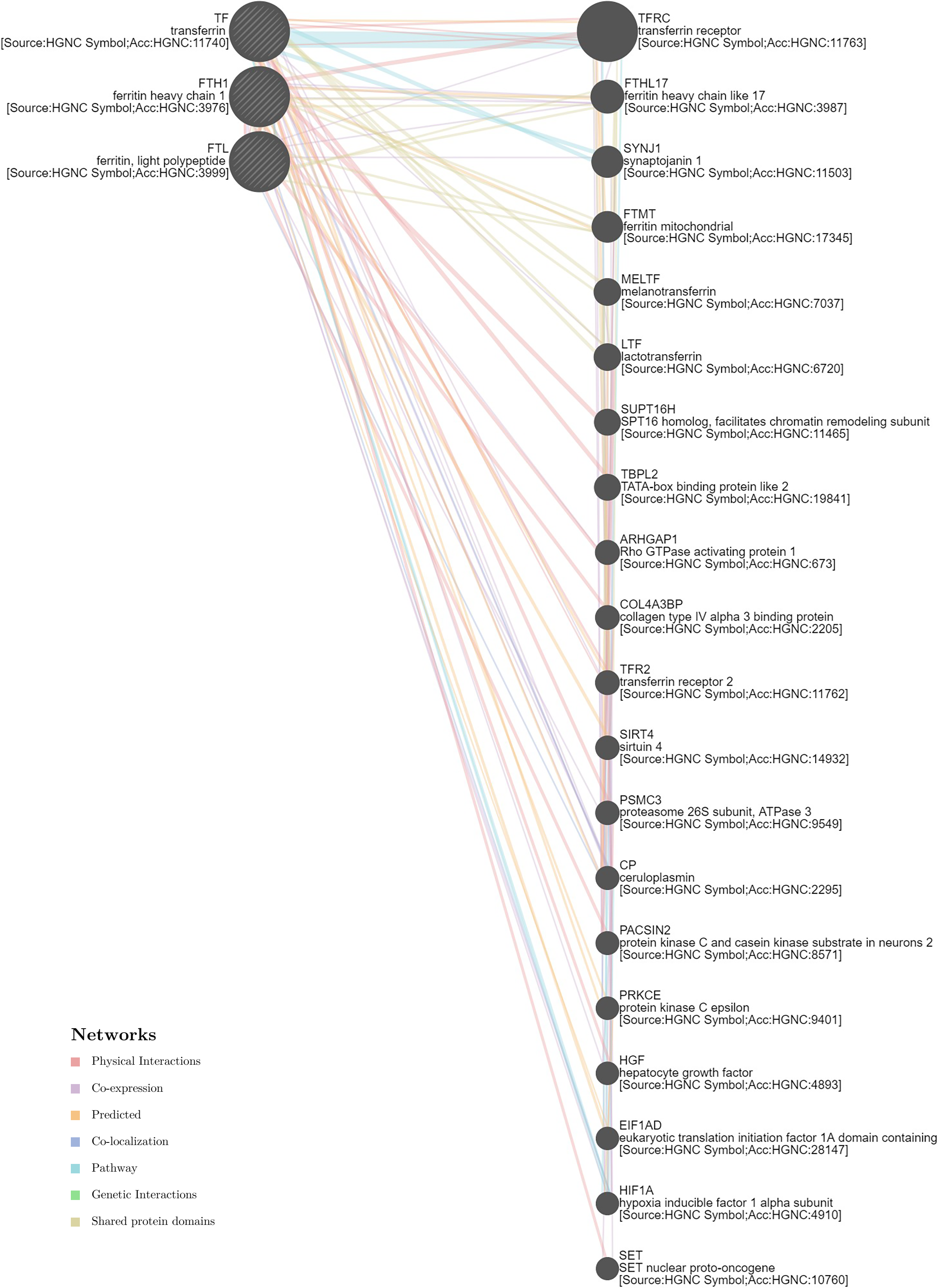

Supplement: Supplementary file 2 [file Image_1.TIF]
