# Supplementary material for: Novel Corneal Protein Biomarker Candidates Reveal Iron Metabolic Disturbance in High Myopia Eyes
Source: Front Cell Dev Biol. 2021 Oct 1;9:689917. doi: 10.3389/fcell.2021.689917 (PMC8517150; doi:10.3389/fcell.2021.689917)
Supplement: Supplementary file 3 [file Data_Sheet_1.docx]

Figure S1. Predictive PPI network of the validated protein biomarker candidates: TF-FTL-FTH1

In the PPI network prediction, the nodes on the left with bias represent the validated protein biomarkers, the nodes on the right stand for the predicted proteins, and lines are mapped for the interactions between proteins. Larger nodes correspond to higher aggregated proteins in the PPI network, and different colors correspond to various functional networks.
